# Surgicel® mimicking a retained appendix on CT: a case report and literature review of diagnostic pitfalls in retained Surgicel® cases

**DOI:** 10.1093/jscr/rjaf388

**Published:** 2025-08-06

**Authors:** Esme Stewart, Matthew McKenna, Rachel Hargest

**Affiliations:** University Hospital of Wales, Heath Park, Cardiff, CF14 4XW, Wales, United Kingdom; University Hospital of Wales, Heath Park, Cardiff, CF14 4XW, Wales, United Kingdom; University Hospital of Wales, Heath Park, Cardiff, CF14 4XW, Wales, United Kingdom

**Keywords:** Surgicel, CT imaging, misdiagnosis, hemostatic agent, radiology, post-op complication

## Abstract

Cross-sectional imaging, particularly computed tomography, is vital in post-operative care but can pose diagnostic challenges. Absorbable hemostatic agents like Surgicel® may mimic abscesses or hematomas, leading to misinterpretation. This review explores these pitfalls and presents a relevant case. 10 studies, including case reports, series, and observational studies, were reviewed to identify imaging characteristics and misdiagnoses of retained Surgicel® across cardiac, neurosurgical, abdominal, and gynecological surgeries. Surgicel® often appears as low-attenuation or gas-containing masses, mimicking complications like abscesses. Misdiagnoses frequently led to unnecessary imaging, treatments, or reoperations. Contributing factors included limited awareness of Surgicel®‘s imaging appearance and potential for contrast enhancement. Misinterpretation of Surgicel® can increase interventions and healthcare costs. Clear communication between surgeons and radiologists about its use and location, along with radiologist awareness and clinical correlation, is key to avoiding misdiagnosis and improving patient care.

## Introduction

Cross-sectional imaging plays a crucial role in the post-operative care of patients who deviate from their expected recovery. While it provides valuable insights, it can introduce diagnostic uncertainty in clinical decision-making. A recognized pitfall in post-operative imaging is the misinterpretation of oxidized regenerated cellulose (Surgicel®), a common absorbable hemostatic agent used in surgery, as pathological fluid collections or abscesses on computed tomography (CT) scans. Failure to consider the full clinical context of these patients can lead to unnecessary interventions, which may expose patients to avoidable risks.

We present a case report and review of the literature, which provides valuable insights into the characteristics and diagnostic challenges of Surgicel®, emphasizing the importance of documenting the used of surgical materials to ensure accurate interpretation of post-operative imaging.

## Case report

We present a case of a 72-year-old male who presented with 5 days of right and lower abdominal pain on the background of ulcerative colitis and previous prostate carcinoma treated with laparoscopic prostatectomy and radiotherapy. Cross-sectional imaging revealed acute appendicitis.

The patient underwent laparoscopic appendicectomy within 24 hours of presentation. Intra-operatively, a locally perforated appendix with a necrotic base was identified, with extensive adhesions to the caecum and terminal ileum, and significant right iliac fossa and pelvic inflammation. Post-radiotherapy necessitated the use of Surgicel® for hemostasis.

**Figure 1:**
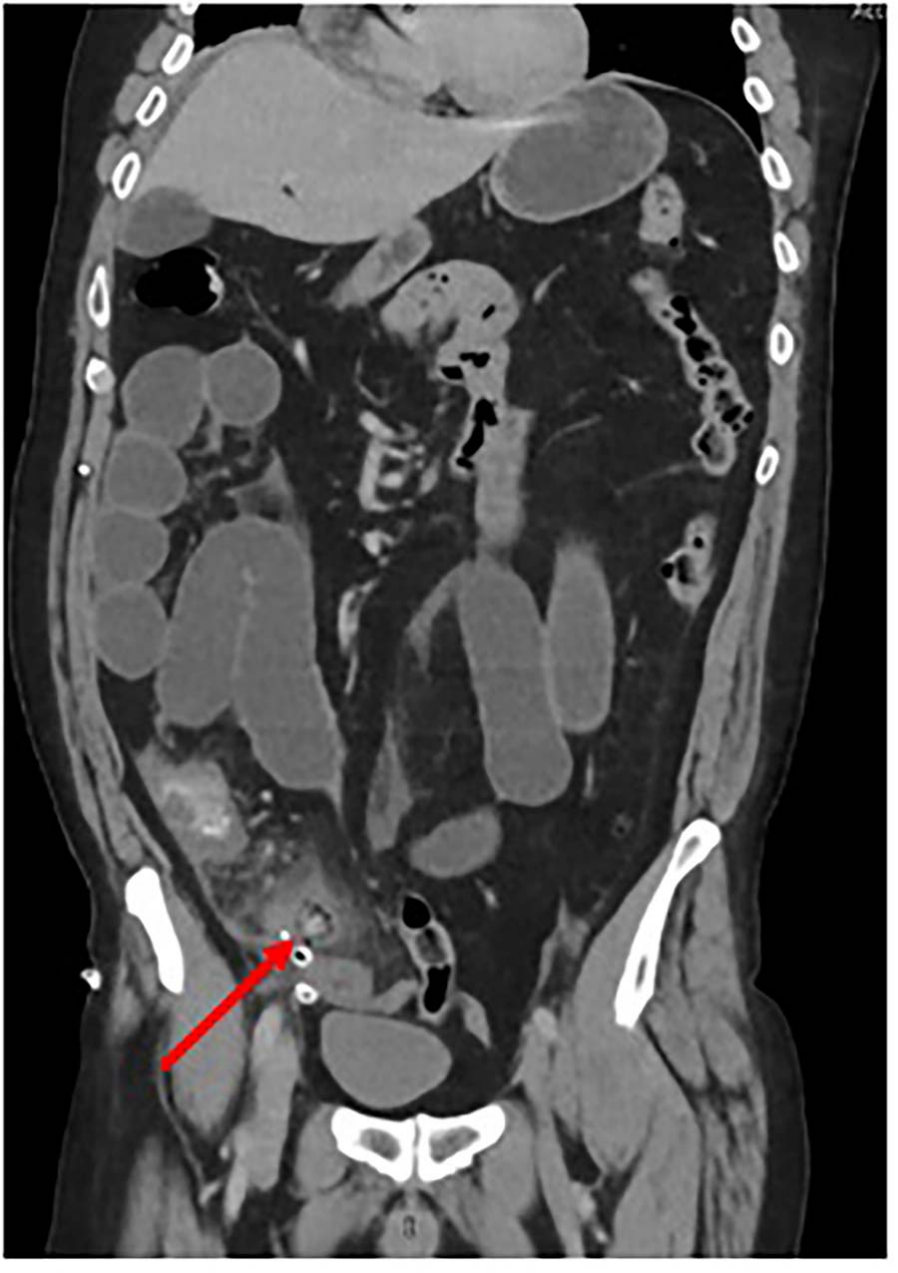
Coronal CT scan depicting Surgicel® as a tubular structure containing gas. (Created by authors)

In the postoperative period, the patient developed an ileus. To exclude a mechanical cause of bowel obstruction, a contrast-enhanced CT abdomen-pelvis was performed. This confirmed an ileus but also reported a ‘Persisting blind ending tubular structure extending from caecum inferior and medially’ and “intraluminal free gas consistent with necrosis” (Fig. 1). Conclusion by the radiologist stated that appearances were consistent with “ongoing appendicitis.”

The decision was made to return to theater for completion appendicectomy. Laparoscopic surgery was unfeasible due to gross bowel dilatation, and so a lower midline laparotomy was performed. The intraoperative findings confirmed a resected appendix with Surgicel® in the surgical bed.

Post-operatively, the patient improved and was discharged on day 10 of admission.

This case is unique as it demonstrates the diagnostic challenge of Surgicel®'s imaging characteristics, leading to the incorrect diagnosis of a retained appendix. The misinterpretation of Surgicel® as a tubular structure on CT highlights the potential for confusion between surgical materials and pathological findings, resulting in re-operation. This emphasizes the need for considering the full clinical context and the presence of materials like Surgicel® when interpreting post-operative imaging.

## Discussion

A narrative review of the currently available literature was performed. The included studies employed various research designs: seven case reports, one case series, one prospective observational study, and one retrospective review. They covered a range of surgical contexts, including cardiac surgery (*n* = 3), neurosurgery (*n* = 2), abdominal surgery (*n* = 2), oncology (*n* = 1), gynecology (*n* = 1), and one study with mixed surgical contexts. Seven of the studies focused on single cases, while three examined multiple cases.

A key finding from the studies reviewed was the various imaging appearances of Surgicel® that often mimic pathological conditions. Azmy [1] described a low-attenuation mass at the site of a resected tumor, while Young [2] reported gas-containing collections within masses of mixed attenuation. Noted fluid attenuation mixed with air locules, and Sandhu *et al.* [3] reported contrast-enhancing space-occupying lesions. These imaging features made it difficult to distinguish Surgicel® from conditions such as abscess, hematoma, and tumor recurrence. Misdiagnosis was a common theme, with post-surgical abscess being the most frequent misdiagnosis, reported in four out of the ten studies.

The studies also identified several factors that contribute to diagnostic challenges, such as gas-fluid collections, unawareness of Surgicel®'s imaging characteristics, contrast enhancement, and mass-like appearances. These elements were particularly problematic in complex surgical contexts, such as post-cardiac surgery, where misinterpretations lead to further interventions.

The misinterpretation of Surgicel® on imaging led to significant clinical consequences in several cases. Unnecessary reoperations were reported in two studies, as detailed in Table 1. Additional diagnostic procedures, such as return to theater [1] and repeated positron emission tomography (PET)/CT scans [4], were performed, and patients were subjected to unnecessary treatments, including a 6-week course of antibiotics for suspected endocarditis [4] and invasive procedures like percutaneous drainage for suspected infected biloma [5]. These unnecessary interventions exposed patients to avoidable risks and resulted in increased healthcare costs.

**Table 1:** Common misdiagnoses seen in reviewed literature (Created by authors)

**Misdiagnosis type**	**Frequency**	**Contributing factors**	**Clinical impact**
Abscess	4/10 studies	Gas-fluid collections, lack of awareness	Potential unnecessary drainage [6, 7]
Tumor recurrence	3/10 studies	Contrast enhancement, mass-like appearance	Unnecessary reoperation
Mediastinitis	2/10 studies	Post-cardiac surgery context	Unnecessary reoperation [8, 9]
Endocarditis	1/10 studies	False-positive PET/CT uptake	Unnecessary antibiotic treatment
Infected biloma	1/10 studies	Complex collection appearance	Consideration of unnecessary drainage

The studies reviewed suggest several strategies to mitigate misinterpretations of Surgicel® and reduce unnecessary interventions. Improved communication between surgeons and radiologists regarding the use and location of Surgicel® was emphasized as a critical strategy. Radiologist education was also considered vital, with several studies highlighting the importance of increasing awareness of Surgicel®'s imaging characteristics to avoid misdiagnosis [2, 10]. Careful correlation of imaging findings with clinical history and surgical context was recommended by Zhang *et al.* [11], and the use of follow-up imaging, such as serial imaging, was suggested to track changes in Surgicel®-related findings [4].

Absorbable Surgicel® is a commonly used hemostatic agent in abdominal surgery, but its high-density radiological appearance can lead to misinterpretation as post-operative collections or hematomas. Literature consistently reports such misdiagnoses, resulting in unnecessary intervention. Furthermore, a prospective study found that 89% of radiologists misinterpreted Surgicel® in post-operative contexts [2] highlighting the importance of awareness of its imaging features.

## Conclusion

This review highlights that gas-fluid collections, contrast enhancement, and mass-like appearances complicate imaging interpretation, particularly in complex cases. Effective communication between surgeons and radiologists about materials like Surgicel® is crucial to prevent misdiagnosis. Additionally, the loss of continuity of care due to shift patterns and working rotas can further contribute to diagnostic challenges. Clear documentation of Surgicel® placement, thorough review of operative notes, and careful clinical correlation are vital to improving diagnostic accuracy and enhancing patient outcomes.

The literature emphasizes the need for greater awareness and collaboration between surgical and radiology teams to reduce the risk of misdiagnosis and unnecessary interventions, improving patient outcomes.

## Statements and declarations

No funding, grants or other support was received. All authors certify that they have no affiliations with or involvement in any organization or entity with any financial interest or non-financial interest in the subject matter or materials discussed in this manuscript.
